# Hypoxia-induced MT2A-tetrameric PKM2 interaction maintains PKM2 activity in a copper-ion-dependent manner

**DOI:** 10.1016/j.cellin.2025.100277

**Published:** 2025-09-24

**Authors:** Ronghui Gao, Qifang Li, Jiahao Guo, Zirou Peng, Chuan Gao, Zirui Zhou, Hankun Hu, Jing Zhang

**Affiliations:** a Department of Urology, Medical Research Institute, Frontier Science Center for Immunology and Metabolism, Zhongnan Hospital of Wuhan University, Wuhan University, Wuhan 430071, Hubei, China; b Department of Pharmacy, Zhongnan Hospital of Wuhan University, School of Pharmaceutical Sciences, Wuhan University, Wuhan 430071, Hubei, China; c Hubei Key Laboratory of Tumor Biological Behavior, Wuhan 430071, Hubei, China

**Keywords:** Mitochondrial translocation, Hypoxia, MT2A, Copper ion, PKM2, Breast cancer growth

## Abstract

Hypoxia is a hallmark of solid tumors associated with tumor malignancy. Mitochondrial metabolic reprogramming is a key step in the process of cellular adaptation to hypoxic stress for tumor growth, but the regulatory mechanisms are not fully understood. In this study, through mitochondrial proteomics analysis, we find that metallothionein-2A (MT2A) is top-ranked among the significantly upregulated proteins in mitochondrial extract in response to hypoxia. Further, we show that hypoxia can induce the mitochondrial translocation of MT2A, a process that is dependent on copper ion. MT2A is highly expressed in hypoxic tumor tissues compared to normoxic ones in breast cancer patients, among whom higher expression of MT2A is associated with a worse prognosis, and it is required for breast tumor growth. Mechanistically, we reveal that copper ion is also essential for hypoxia-induced mitochondrial translocation of Pyruvate kinase M2 (PKM2), and facilitates the interaction of MT2A with the tetrameric form of PKM2 to maintain its activity, thereby promoting glycolysis and oxidative phosphorylation. Thus, our findings reveal the MT2A-copper-PKM2 axis as a potential therapeutic target to treat breast cancer.

## Highlights

• MT2A is the most significantly upregulated protein on mitochondria under hypoxia.
• MT2A contributes to breast cancer cell growth.
• Hypoxia induces the MT2A-tetrameric PKM2 interaction.
• MT2A sustains mitochondrial localization and catalytic activity of PKM2 under hypoxia via copper ion.

## Graphical abstract

**Figure:**
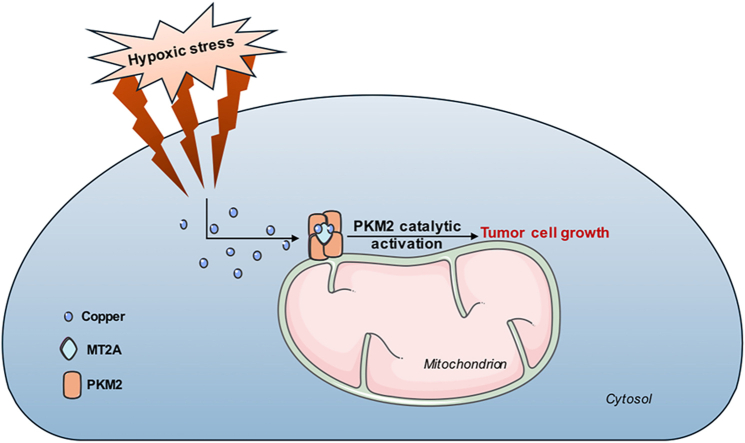

## 1 Introduction

Hypoxia is a key factor in the tumor microenvironment, which is associated with tumor malignancy, drug resistance and therefore higher mortality rates (Infantino et al., 2021; McKeown, 2014). Hypoxia-inducible factor (HIF) is well-known as a major regulator in cellular adaptation to hypoxia. Under normoxia, HIFα is hydroxylated by the prolyl hydroxylases EglNs, and then targeted by the von Hippel Lindau (VHL) E3 ligase complex for ubiquitination and subsequent proteasomal degradation (Kaelin & Ratcliffe, 2008). While the prolyl hydroxylation of HIFα is suppressed under hypoxia, leading to HIFα accumulation and heterodimerization with HIF1β (ARNT), which then binds the hypoxia response elements (HREs) to promote transcription of genes essential for cancer cell proliferation, angiogenesis, and etc (Lee et al., 2021; Losman et al., 2020; Semenza, 2012; Yang & Kaelin, 2001).

Numerous lines of evidence support that sophisticated interaction networks beyond the HIF pathway contribute to cellular adaptation to hypoxia (Batie et al., 2019; Chakraborty et al., 2019; Gao et al., 2025; Guo et al., 2016; Hu et al., 2020; Jiang et al., 2023; Lee et al., 2015, 2020; Liu et al., 2020; Qian et al., 2019; Zhang et al., 2018). Among those, mitochondrial metabolism is an important mechanism that allows cellular adaptation to hypoxic stress in a HIF-dependent and -independent manner (Lee et al., 2016, 2020). Mitochondrion is a dynamic double membrane-bound organelle with multiple channels and transporters that transport different metal ions such as Ca^2+^, K^+^, Na^+^, Mg^2+^, Zn^2+^, Fe^2+^/Fe^3+^ and Cu^+^/Cu^2+^. Many evidences suggest that the transport of metal ions is critical for mitochondrial function and cellular metabolism, and serves as a potential target for the treatment of metabolic diseases, such as cancer (Tsvetkov et al., 2022b; Wang et al., 2021).

Metallothionein-2A (MT2A), a cysteine-rich and metal-binding protein, is a hypoxia-inducible gene (Dubé et al., 2011; Yamasaki et al., 2007). Its induction in response to hypoxia maintains the balance of metal ions such as copper and zinc, protects cells from apoptosis by reducing oxidative stress, and thus serves as a critical component of cellular adaptation to hypoxic stress (Dubé et al., 2011; Ling et al., 2016; Yamasaki et al., 2007). But, the underlying mechanism of how hypoxia-induced MT2A regulates mitochondrial metabolism remains poorly understood. In this study, we unveil that MT2A accumulates on mitochondria under hypoxia and interacts with tetrameric PKM2 to maintain its mitochondrial localization and catalytic activity in a copper-ion-dependent manner, thus contributing metabolic adaptation to hypoxic stress.

## 2 Results

### 2.1 MT2A accumulates on mitochondria under hypoxia dependent on copper ion

**Fig. 1:**
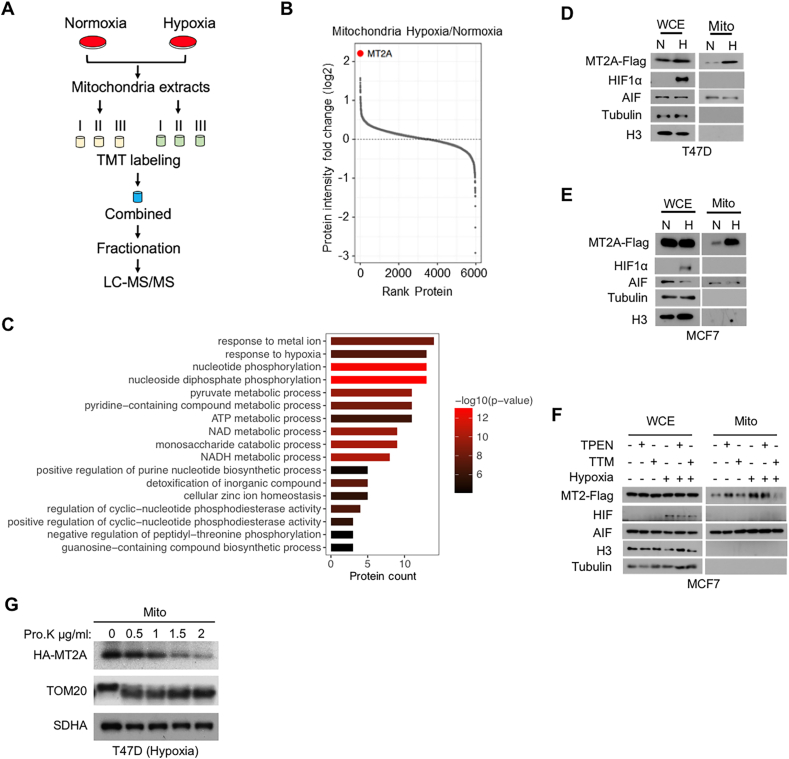
**MT2A accumulates on mitochondria under hypoxia involving copper.(A)** Schematic representation of the mitochondrial protein profile by mass spectrometry. **(B)** Mitochondrial proteome rank ordering map of T47D cells pre-treated with hypoxia (1% O_2_ for 24 h) versus normoxia. MT2A is highlighted in red. **(C)** GO pathway enrichment analysis of mitochondrial proteome in T47D cells. -log_10_ (*P*-value) > 4 was used as a cutoff. **(D**–**E)** Immunoblots of whole cell (WCE) and mitochondrial (Mito) extracts from T47D cells (D) and MCF7 cells (E), all treated with hypoxia (H, 1% O_2_ for 24 h) or normoxia (N). **(F)** Immunoblots of hypoxic (1% O_2_ for 24h) or normoxic MCF7 cell extracts from whole cell (WCE), mitochondria (Mito). MCF7 cells pre-treated with TPEN (2.5 μM) or TTM (2.5 μM) for 24 h. **(G)** Immunoblots of hypoxic (1% O_2_ for 24 h) T47D mitochondrial extract (Mito) treated with indicated concentrations of proteinase K for 30 min.

To unravel the mechanisms of how breast cancer cells adapt to hypoxic environment via mitochondria, we cultured breast cancer cell line T47D under hypoxia or normoxia for 24 h and then isolated mitochondria to characterize the proteome using quantitative proteomics approach (Fig. 1(A)). We identified about 6000 proteins in mitochondrial extracts and sorted them by hypoxia versus normoxia. Among these, MT2A is the most significantly upregulated protein in response to hypoxia (Fig. 1(B)). Gene ontology (GO) pathway enrichment analysis of these proteins showed that the response to metal ion pathway was ranked top one (Fig. 1(C)). MT2A is a metal ion binding protein that regulates intracellular metal ion homeostasis (Krizkova et al., 2018), so the above results suggested that MT2A may function as a metal ion regulatory factor by translocating to mitochondria in response to hypoxia.

Previous studies have reported that MT2A is a hypoxia-inducible gene (Dubé et al., 2011; Yamasaki et al., 2009), and thus hypoxia-induced transcriptional upregulation of MT2A could contribute to its mitochondrial accumulation. To investigate whether the potential induction of MT2A mitochondrial accumulation under hypoxia is independent of its transcriptional upregulation, we performed mitochondrial fractionation with MCF7 or T47D cells expressing exogenous MT2A-Flag, which showed that hypoxia led to mitochondrial accumulation of exogenous MT2A, without significant effect on its total levels in cells (Fig. 1(D) and (E)). These data suggest that MT2A can translocate to mitochondria in response to hypoxia. MT2A can bind to zinc ion or copper ion to maintain intracellular zinc or copper homeostasis to reduce cellular metal toxicity (Moffatt & Denizeau, 1997). We treated breast cancer cells with TPEN (a chelator of zinc ion) or TTM (a chelator of copper ion) for 24 h. The results showed that chelation of copper ion, but not zinc ion, attenuated mitochondrial accumulation of MT2A under hypoxia, while the total protein levels of MT2A were not affected (Fig. 1(F)). Proteinase K digestion showed that the majority of MT2A was localized on the mitochondrial outer membrane (Fig. 1(G)). In conclusion, these results indicated that hypoxia leads to the translocation of MT2A to mitochondria, and this process requires copper ion.

### 2.2 MT2A is pathologically relevant to breast cancer

MT2A is a hypoxia-inducible gene (Dubé et al., 2011; Yamasaki et al., 2007). Herein, we have showed that MT2A also undergoes translocation to mitochondria in response to hypoxia, implying a positive correlation between its total cellular expression levels and mitochondrial accumulation levels. These coordinated changes suggest that MT2A may function as a hypoxia-responsive molecular effector, integrating transcriptional induction with subcellular translocation to mediate tumor adaptive responses in the hypoxic tumor microenvironment. At the current stage, in order to investigate the pathological relevance of hypoxia-responsive MT2A in breast cancer, we analyzed the human breast cancer single-cell RNA sequencing (scRNA-seq) dataset (Wu et al., 2021b) to examine the correlation between MT2A expression patterns and tumor hypoxic status. This analysis may reflect a corresponding correlation between MT2A mitochondrial levels and tumor hypoxic status.

**Fig. 2:**
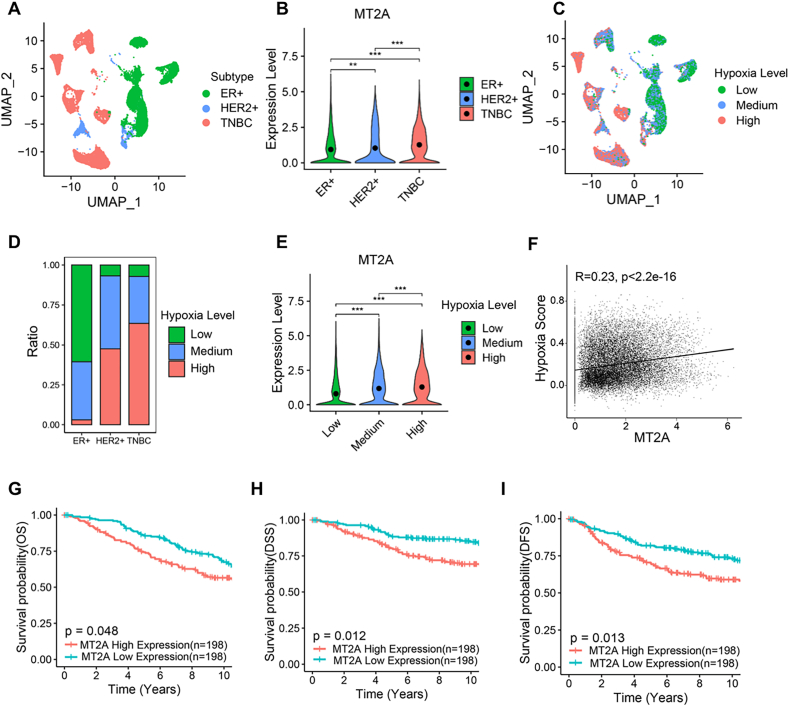
**MT2A is pathologically relevant to breast cancer.(A**–**B)** UMAP visualization of different breast cancer subtypes in human breast cancer single-cell data and colored by clinical subtype (A), right side is violin plot of MT2A gene expression (B). **(C**–**D)** UMAP visualization of different breast cancer subtypes in human breast cancer single-cell data and colored by hypoxia level (C), and relative proportions of hypoxia level (D). **(E)** Violin plots of MT2A expression at different hypoxia levels. **(F)** Dot plots of MT2A correlation with hypoxia score. **(G**–**I)** Kaplan-Meier plots of survival data for patients with different breast cancer subtypes in METABRIC database stratified by MT2A gene expression levels. Overall Survival (OS) (E), Disease Specific Survival (DSS) (F), Disease Free Survival (DFS) (G), Patient numbers are shown. All error bars represent SD, two-tailed Student *t*-test (∗, *P* < 0.05; ∗∗, *P* < 0.01; ∗∗∗, *P* < 0.001; ns, denotes no significance). (A)-(F) based on human breast cancer single-cell data(Wu et al., 2021a).

We clustered cancer cells from patients with different subtypes of breast cancer (Fig. 2(A)), and found that cancer cells from TNBC patients had the highest expression levels of MT2A (Fig. 2(B)). Next, we used the Buffa gene signature (Buffa et al., 2010) for reflecting hypoxia levels to characterize the grade of hypoxia status in different breast cancer subtypes, and the results showed that the TNBC subtype had the highest hypoxia level (Fig. 2(C) and (D)). And, the expression levels of MT2A was positively correlated with hypoxia scores (Fig. 2(E) and (F)). Previous study showed that TNBC showed the worst prognosis in breast cancer, this analysis indicated that higher expression of MT2A might be associated with a worse prognosis. Indeed, through analysis of METABRIC database, we found that higher expression of MT2A was associated with worse prognosis (Fig. 2(G)–(I)). Altogether, these data suggested the pathological relevance of MT2A in breast cancer.

### 2.3 MT2A contributes to breast cancer cell growth

**Fig. 3:**
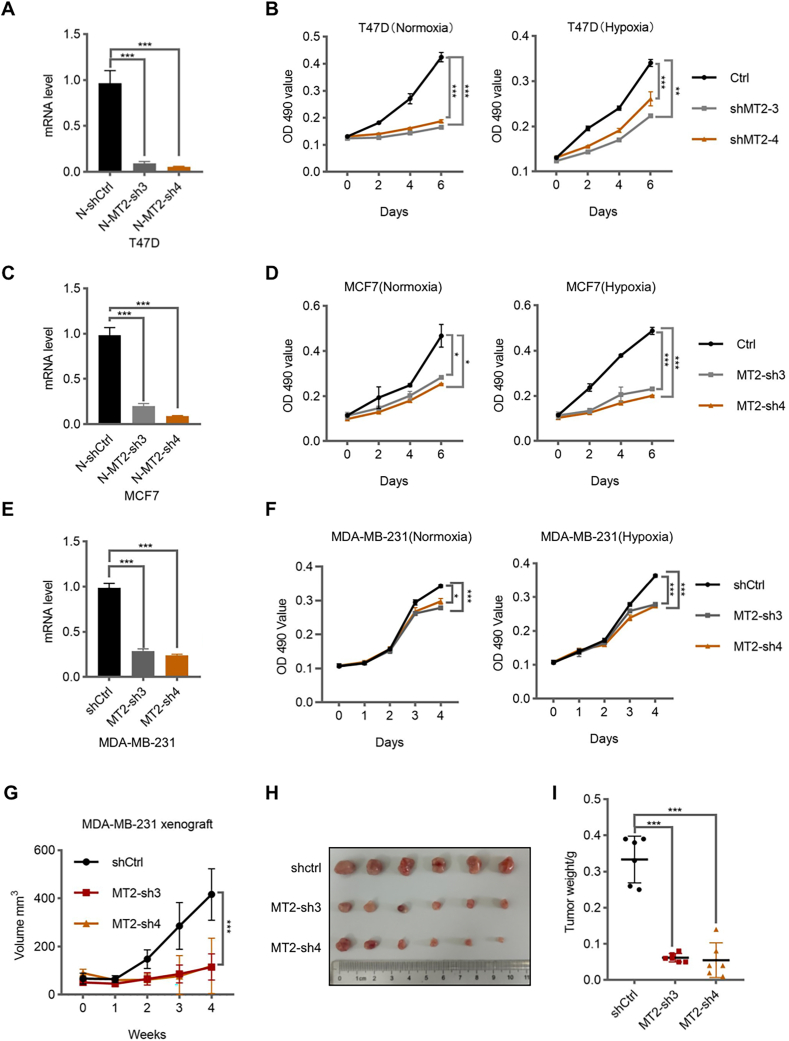
**MT2A is required for breast cancer cell growth.(A, C, E)** MT2A mRNA level in T47D cells (A), MCF7 cells (C), MDA-MB-231 cells (E) determined by real time quantitative PCR (RT-qPCR). **(B, D, F)** MTT assays from T47D cells (B), MCF7 cells (D), MDA-MB-231 cells (F) cell lines with MT2A depletion under hypoxia (1 % O_2_) or normoxia conditions. **(G**–**I)** Mouse xenograft experiments with MDA-MB-231 cell line with MT2A depletion. Tumor growth curves (G) and tumor weights (I) were calculated, and gross tumors were presented (H) (*n* = 6 mice per group). All error bars represent SD, two-tailed Student *t*-test (∗, *P* < 0.05; ∗∗, *P* < 0.01; ∗∗∗, *P* < 0.001).

To investigate the function of MT2A on breast cancer. We examined the effect of MT2A in breast cancer cell proliferation. We found that depletion of MT2A inhibited the breast cancer cell proliferation in T47D, MCF7 and MDA-MB-231 cell lines (Fig. 3(A)–(F)). These results indicated that MT2A plays an essential role in breast cancer cell proliferation *in vitro*.

To validate this conclusion *in vivo*, we knocked down MT2A in MDA-MB-231 cells and orthotopically injected these MT2A-depleted MDA-MB-231 cells into the mammary gland of nude mice. By monitoring tumor growth every third day by caliper, we found that depletion of MT2A also inhibited breast cancer growth *in vivo* (Fig. 3(G)–(I)). In conclusion, these results suggested that MT2A is essential for breast cancer growth.

### 2.4 MT2A interacts with tetrameric PKM2 under hypoxia

**Fig. 4:**
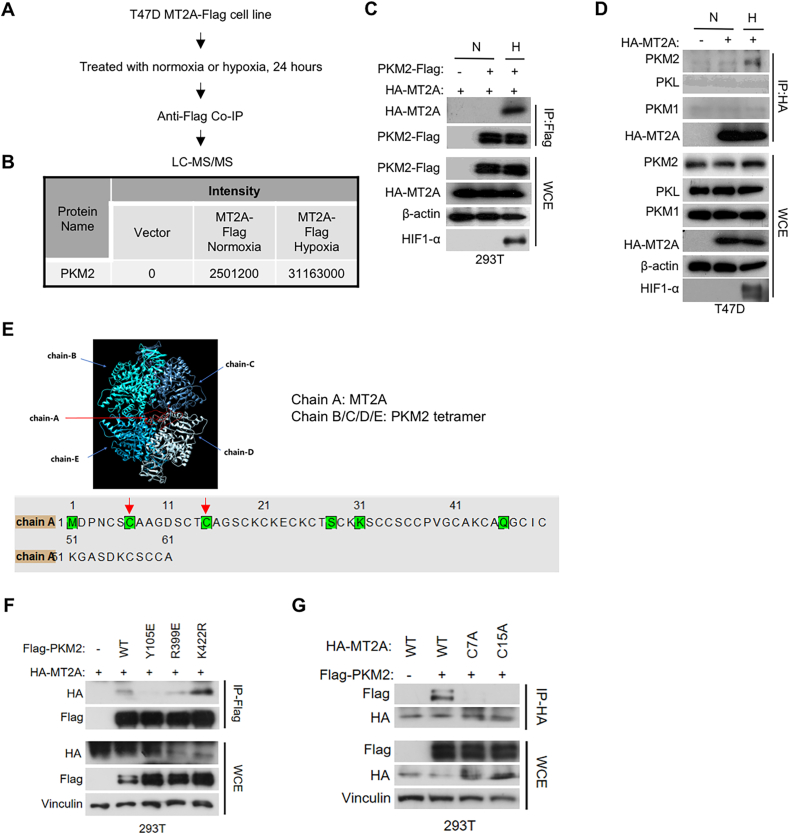
**MT2A interacts with tetrameric PKM2 under hypoxia.(A**–**B)** Schematic representation of strategy for identification of MT2A-interacting proteins in T47D cells exposed to hypoxia (1% O_2_ for 24 h) versus normoxia by mass spectrometry (A). Below schematic is the sequence intensity of PKM2 from mass spectrometry analysis (B). **(C)** Immunoblots (IB) of whole cell extracts (WCE) and immunoprecipitations (IP) of 293T cells transiently transfected with HA-MT2 and PKM2-Flag followed by treatment with hypoxia (1% O_2_ for 24 h) or normoxia conditions. **(D)** Immunoblots (IB) of whole cell extracts (WCE) and immunoprecipitations (IP) from T47D cells stably expressing HA-MT2 followed by treatment with hypoxia (1% O_2_ for 24 h) or normoxia. **(E)** Interaction model of MT2A with PKM2 predicted with Alphafold2, the chain-A is MT2A (point out with red arrows), chain-B/C/D/E are PKM2, and at the bottom is the predicted interaction site of chain-A with tetrameric PKM2. The confidence level of the demonstrated model is 0.908 and the threshold is set at 0.75. **(F)** Immunoblots (IB) of whole cell extracts (WCE) and immunoprecipitations (IP) of 293T cells transiently transfected with HA-MT2 and PKM2-Flag or PKM2 point mutants (Y105E, R399E, K422R). **(G)** Immunoblots (IB) of whole cell extracts (WCE) and immunoprecipitations (IP) of 293T cells transiently transfected with HA-MT2A or MT2A point mutants (C7A, C15A) and PKM2-Flag followed by treatment with hypoxia (1% O_2_ for 24 h) conditions. All error bars represent SD, two-tailed Student *t*-test (∗, *P* < 0.05; ∗∗, *P* < 0.01; ∗∗∗, *P* < 0.001; ∗∗∗∗, *P* < 0.0001).

Given that MT2A carrying copper can regulate protein activity, we determined how it functions by interacting with potential proteins on mitochondria. We performed MT2A-Flag immunoprecipitation-mass spectrometry (IP-MS) in T47D cells under normoxia versus hypoxia (Fig. 4(A)). By analyzing the IP-MS data, we identified PKM2 as MT2A-interacting protein, which showed much stronger interaction in hypoxia than in normoxia (Fig. 4(B)). Co-immunoprecipitation (Co-IP) assays confirmed that MT2A could interact with PKM2 under hypoxia (Fig. 4(C) and (D)), while showed no interactions with other pyruvate kinase isozymes, such as PKM1 and PKL (Fig. 4(D)).

PKM is a rate-limiting enzyme in final step of glycolysis, catalyzing the conversion of phosphoenolpyruvate to pyruvate (Noguchi et al., 1986). It exists in a dynamic population of monomer, dimer or tetramer in cells. The tetrameric PKM2 exerts the pyruvate kinase catalytic activity prone to localized on mitochondria, while the dimeric PKM2 acts as a transcriptional coactivator or protein kinase in nucleus (Christofk et al., 2008; Gao et al., 2012; Liang et al., 2024; Luo et al., 2011; Wang et al., 2015; Yang et al., 2011). Therefore, we predicted how MT2A might interact with these different forms of PKM2 with Alphafold2, and found that MT2A could interact with the PKM2 tetramer with the highest confidence (cutoff = 0.908) in the prediction models (Fig. 4(E)). Then, we constructed three different mutants of PKM2 including Y105E (tending to form monomer), R399E (tending to form dimer) and K422R (tending to form tetramer) (Wang et al., 2015) and examined their interactions with MT2A, respectively. Consistent with the Alphafold2 prediction, the Co-IP assays showed that the tetrameric PKM2 interacted with MT2A in the most efficient way (Fig. 4(F)).

Meanwhile, Alphafold2 prediction showed that there were six amino acid residues on MT2A that contacted with PKM2 tetramer, among those sites, the cysteine 7 and cysteine 15 are able to bind with metal ion for MT2A metal ion-binding ability, such as binding with copper ion (Ge et al., 2022; Ruttkay-Nedecky et al., 2013) (Fig. 4(E)). Thus, we wondered whether MT2A-mediated metal ion-binding could affect its interaction with PKM2, and found that mutation of either cysteine 7 or cysteine 15 to alanine attenuated its interaction with PKM2 under hypoxia, suggesting that the MT2A-PKM2 interaction may require metal ion (Fig. 4(G)). Collectively, these data suggested that MT2A relies on its metal-binding sites cysteine 7 and cysteine 15 to interact with PKM2 tetramer.

### 2.5 MT2A sustains mitochondrial localization and catalytic activity of PKM2 under hypoxia in a copper-ion-dependent manner

**Fig. 5:**
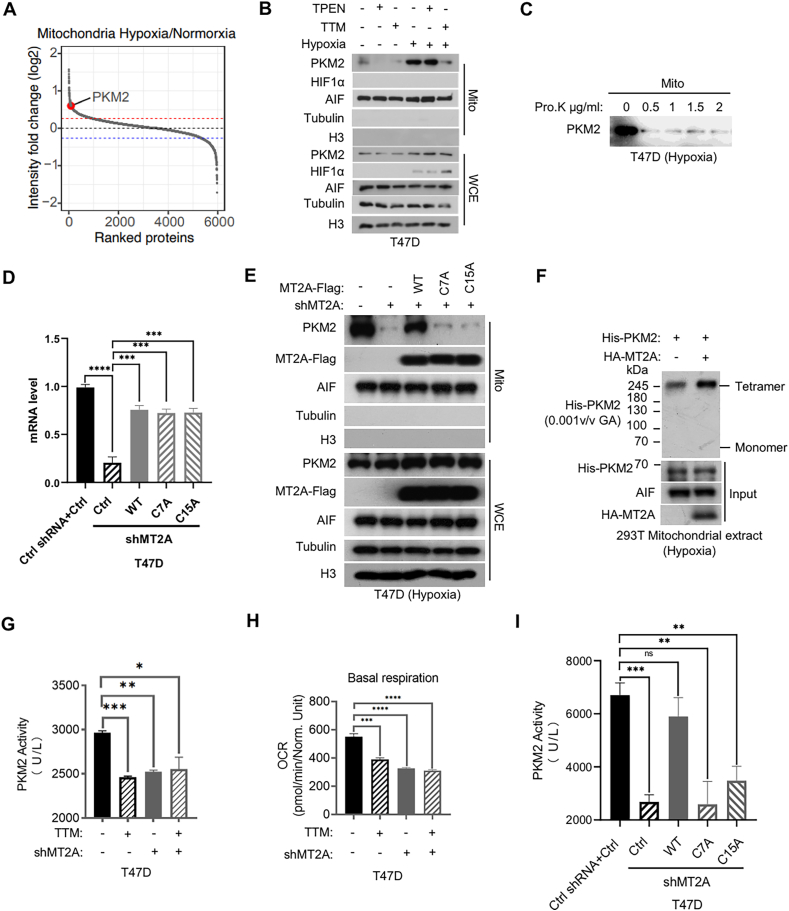
**MT2A sustains mitochondrial localization and catalytic activity of PKM2 under hypoxia in acopper-ion-dependentmanner.(A)** Mitochondrial proteome rank ordering map in Fig. 1(B), and PKM2 is highlighted in red. **(B)** Immunoblots of hypoxic (1% O_2_ for 24 h) or normoxic T47D cells extracts from whole cell (WCE), mitochondria (Mito). T47D cells pre-treated with TPEN (2.5 μM) or TTM (2.5 μM) for 24 h. **(C)** Immunoblots of mitochondrial extract (Mito) from Fig. 1(G). **(D)** Real time quantitative PCR (RT-qPCR) measurements of MT2A mRNA levels in T47D cells infected with control vector, MT2A-Flag, or MT2A mutants (C7A, C15A) followed by another infection with shCtrl or shMT2A. **(E)** Immunoblots of whole cell (WCE) and mitochondrial (Mito) extracts from hypoxic (1% O_2_ for 24 h) T47D cells as described in (D). **(F)** Mitochondrial extract from hypoxic (1% O_2_ for 24 h) 293T cells transfected with HA-Vector or HA-MT2A were incubated with purified recombinant His-PKM2 protein, followed by cross-linking with 0.001 (*V*/*V*) GA (glutataldehyde) and consequently detected by immunoblots. **(G)** PKM2 activity assays from hypoxic (1% O_2_ for 24 h) T47D cells infected with shCtrl, shMT2A, treated with TTM (2.5 μM), or with shMT2A plus TTM treatment for 24 h. **(H)** Quantification of basal mitochondrial oxidative phosphorylation from T47D cells as described in (G). **(I)** PKM2 activity assays from hypoxic (1% O_2_ for 24 h) T47D cells as described in (D). All error bars represent SD, two-tailed Student *t*-test (∗, *P* < 0.05; ∗∗, *P* < 0.01; ∗∗∗, *P* < 0.001; ∗∗∗∗, *P* < 0.0001).

Given that tetrameric PKM2 tends to localized on mitochondria (Liang et al., 2024), hypoxia induces mitochondrial translocation of MT2A and leads to MT2A-PKM2 tetramer interaction in a metal-ion-dependent manner, we next sought to determine whether hypoxia can also induce the mitochondrial accumulation of PKM2. Similar to MT2A, mitochondrial proteomics analysis showed that PKM2 also accumulated on mitochondria under hypoxia (Fig. 5(A)). And mitochondrial extraction confirmed that hypoxia could induce mitochondrial accumulation of PKM2 (Fig. 5(B)). TTM, but not TPEN, attenuated this process (Fig. 5(B)), suggesting that hypoxia-induced mitochondrial accumulation of PKM2 also depends on copper ion. Similar to MT2A, proteinase K digestion showed that the majority of PKM2 was also localized on the mitochondrial outer membrane (Fig. 5(C)). MT2A depletion could inhibit the accumulation of PKM2 on mitochondria in hypoxia, MT2A WT, but not C7A or C15A mutants, could rescue the PKM2 mitochondrial accumulation (Fig. 5(D) and (E)), suggesting that PKM2 mitochondrial localization requires its interaction with MT2A. Taken together, these data suggest that hypoxia-induced MT2A-tetrameric PKM2 interaction occurs on mitochondria in a copper-ion-dependent manner.

We therefore hypothesized that MT2A carrying copper ion to interact with PKM2 in hypoxia is used for stabilizing tetrameric form of PKM2, thereby maintaining its pyruvate kinase catalytic activity, we thus examined the effect of MT2A or copper ion on the PKM2 activity. First, through *in vitro* protein cross-linking experiment, we examined PKM2 oligomerization, since the tetrameric PKM2 exerts the pyruvate kinase catalytic activity (Liang et al., 2024). This assay showed that mitochondrial MT2A from hypoxic cells could promote tetramer formation of the purified recombinant His-PKM2 *in vitro* (Fig. 5(F)), suggesting that MT2A can enhance PKM2 enzymatic activity. Then, we found that either depletion of MT2A or chelation of copper ion by TTM treatment could attenuate PKM2 activity, but MT2A depletion plus TTM treatment failed to decrease its activity even lower than any of these treatments (Fig. 5(G)), indicating that MT2A and copper ion regulate PKM2 activity in the same signaling axis. Consistently, depletion of MT2A and chelation of copper ion could also inhibit the mitochondrial oxidative phosphorylation (Fig. 5(H)). Consistently, MT2A WT, but not C7A or C15A mutant, could rescue the decreased PKM2 activity caused by MT2A depletion (Fig. 5(I)). Taken together, these data suggest that MT2A accumulates on mitochondria under hypoxia to interact with tetrameric PKM2 for sustaining its mitochondrial localization and catalytic activity in a copper-ion-dependent manner.

### 2.6 The expression levels of MT2A and PKM2 are highly correlated in breast cancer

**Fig. 6:**
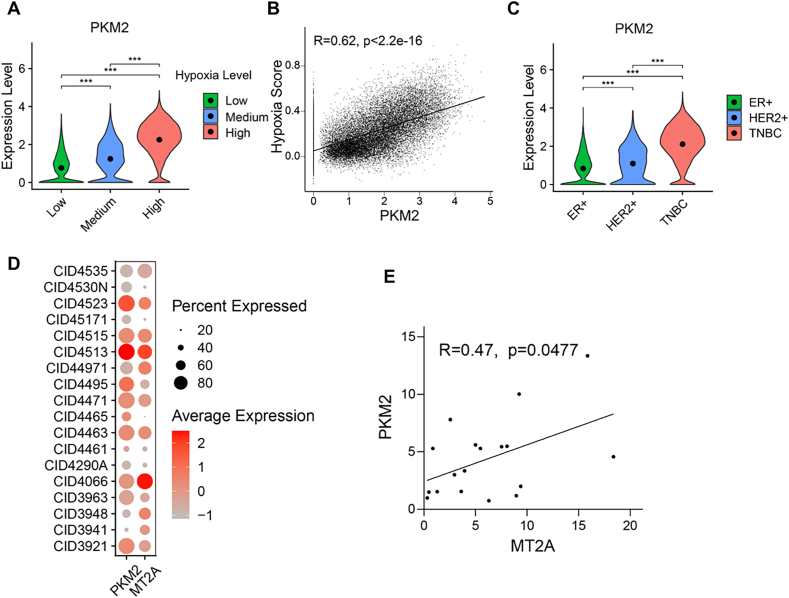
**The expression levels of MT2A and PKM2 are highly correlated in breast cancer.(A)** Violin plots of PKM2 expression at different hypoxia levels. **(B)** Dot plots of PKM2 correlation with hypoxia score. **(C)** Violin plot of PKM2 expression in different breast cancer subtypes. **(D)** Dot plot of MT2A and PKM2 gene expression in breast cancer patients, the size of the circle represents the number of expressed cells, and the color shade represents the degree of expression. **(E)** Dot plot of the correlation between MT2A and PKM2. All error bars represent SD, two-tailed Student *t*-test (∗∗∗, *P* < 0.001). All data based on human breast cancer single-cell data (Wu et al., 2021a).

PKM2 is also a hypoxia-inducible gene (Luo et al., 2011), and, similar to MT2A, we have found that PKM2 also translocates to mitochondria under hypoxia. Accordingly, we extended this analytical framework of MT2A to investigate the pathological relevance of hypoxia-responsive PKM2 in breast cancer with the human breast cancer scRNA-seq dataset (Wu et al., 2021b) to examine the correlation between PKM2 expression patterns and tumor hypoxic status. This analysis showed that, similar to MT2A, the expression levels of PKM2 were positively correlated with hypoxia scores in breast cancer (Fig. 6(A) and (B)). The cancer cells from TNBC patients also had the highest expression levels of PKM2 (Fig. 6(C)).

Given that both MT2A and PKM2 are hypoxia-inducible genes and undergo mitochondrial translocation in response to hypoxia, we speculated that the expression levels of MT2A and PKM2 may exhibit a strong correlation in cancer. Therefore, we investigated the correlations between MT2A and PKM2 based on human breast cancer scRNA-seq dataset (Wu et al., 2021a). Indeed, we found that the expression levels of MT2A showed a strong positive correlation with those of PKM2 in tumor cells across different breast cancer patients (Fig. 6(D) and (E)). These data suggest that the expression levels of MT2A and PKM2 are highly correlated in breast cancer patients, implying the pathological relevance of the MT2A-PKM2 axis in cancer.

## 3 Discussion

Hypoxic microenvironment is a key feature of solid tumors that can drive cancer malignancy (Infantino et al., 2021). As the primary oxygen-sensing organelle in tumor cells, mitochondria respond to hypoxic stress by signaling transduction to reprogram metabolism (Jiang et al., 2023). Here, we provide a new perspective on how cancer cells adapt to hypoxic microenvironment through mitochondrial MT2A. Our study elucidates a copper-mediated mitochondrial translocation mechanism underlying breast cancer cell adaptation to hypoxia. Mechanistically, we identify a hypoxia-responsive signaling axis where copper orchestrates MT2A trafficking to mitochondria, thereby modulating PKM2-dependent metabolic adaptation. This demonstration of the MT2A-copper-PKM2 regulatory axis provides a therapeutic framework targeting metal chaperone-mediated metabolic plasticity in hypoxic tumors.

MT2A is a hypoxia-inducible gene, positively regulated by HIF-1 pathway, which links MT2A to the master regulatory network controlling hypoxic stress adaptation (Yamasaki et al., 2007). As a metal-binding protein, MT2A maintains metal ion homeostasis by chelating and detoxifying heavy metal ions released during hypoxic stress, while preserving the balance of essential ions (e.g., copper and zinc) (Dubé et al., 2011; Ling et al., 2016; Yamasaki et al., 2007). MT2A upregulation is frequently observed in tumors, where it correlates with poor patient prognosis (Ling et al., 2016). In the hypoxic tumor microenvironment, MT2A supports cancer cell survival by neutralizing oxidative stress and contributing to chemotherapy resistance (Krizkova et al., 2018; Moffatt & Denizeau, 1997). Recently, a published study has demonstrated that HIF-1α-induced MT2A accumulation can sequester mitochondrial copper to blunt apoptotic signaling, thereby promoting cancer cell resistance to cell death (Yang et al., 2025). Here, our work uncovers a previously unrecognized role of mitochondrial MT2A in metabolic adaptation to hypoxia. We reveal that MT2A can translocate to mitochondria upon hypoxia, where it interacts with tetrameric PKM2 via copper ions, thereby sustaining the mitochondrial localization and catalytic activity of PKM2. This finding reveals a new regulatory mechanism by which MT2A couples copper homeostasis to metabolic adaptation, extending our understanding of MT2A's role in direct control of a central metabolic enzyme (i.e., PKM2) under hypoxia.

Copper is a cofactor for multiple key mitochondrial enzymes and is essential for the regulation of mitochondrial function and cellular metabolism, Imbalance in intracellular copper homeostasis impairs cellular function and contributes to the development of cancer (Festa & Thiele, 2011; Wang et al., 2021). Excess copper impairs mitochondrial function, leading to cytotoxicity and cuproptosis (Tsvetkov et al., 2022a). The cuproptosis resistance has been characterized as a feature of hypoxic cells in solid tumor (Yang et al., 2025). Inhibition of cancer cell growth by reducing or elevating intracellular copper levels has been proposed as potential cancer therapies. Due to the toxicity and poor therapeutic efficacy of current copper chelators, these clinical applications remain challenging, and new auxiliaries, such as mitochondrial copper-depleting nanoparticle (CDN), show the possibility for clinical application (Cui et al., 2021). But the underlying mechanisms remain elusive. Here, we found that hypoxia can induce MT2A accumulation on mitochondria and its interaction with tetrameric PKM2 in a copper-ion-dependent manner, thereby maintaining the PKM2 activity. Our findings establish a copper-dependent MT2A trafficking to mitochondria as a therapeutic target that simultaneously sabotage metal homeostasis and metabolic reprogramming in breast malignancies.

PKM2 is upregulated in multiple cancer cells and contributes to tumor progression (Israelsen et al., 2013). PKM2 can translocate to mitochondria under oxidative stress and inhibit apoptosis by stabilizing B-cell lymphoma 2 (Bcl2) (Liang et al., 2017). It can also undergo mitochondrial translocation under nutritional stress, such as glucose starvation, to promote cell survival and tumor growth (Qi et al., 2019). Also, cancerous inhibitor of protein phosphatase 2A (CIP2A) enhances oxidative phosphorylation by promoting tetramer PKM2 formation and redirecting PKM2 to mitochondria, thereby promoting tumor progression (Liang et al., 2024). Collectively, these studies suggest that blocking PKM2 mitochondrial translocation or function has the potential to treat cancer. Our study for the first time reveals that the PKM2 activity is dependent on MT2A and copper ion under hypoxia; Alphafold2 predication along with mutagenesis and Co-IP approach to valid their interacting sites suggests that MT2A carrying copper ion may structurally stabilize tetrameric form of PKM2 and maintain its pyruvate kinase catalytic activity. Thus, we illustrate a regulatory mechanism that cancer cell becomes adaptive to hypoxic microenvironment by copper-ion-mediated PKM2 activation, highlighting an essential role of PKM2-dependent metabolic plasticity during cancer progression, and revealing a protein-protein interaction pocket in the central of tetrameric PKM2 that could be targeted by a small molecule inhibitor for cancer treatment.

## 4 Materials and methods

### 4.1 Cell culture and reagents

T47D (ATCC HTB-133) was maintained in RPMI (C11875500BT) medium supplemented with 10% fetal bovine serum and 1% penicillin-streptomycin. MDA-MB-231 (ATCC HTB-26), MCF7 (ATCC HTB-22), and 293T (ATCC CRL-3216) were cultured in DMEM (C11995500BT) containing 10% fetal bovine serum and 1% penicillin-streptomycin. All cells were cultured at 37 °C with 5% CO_2_. TPEN (S6962) from Selleck. TTM (15060-5S-6) from Sigma. The above reagents were used at concentrations previously described (Fang et al., 2019; Zhu et al., 2017). Hypoxia treatment in hypoxia workstation (Baker Ruskinn's InvivoO_2_ 400).

### 4.2 Western blot analysis and antibodies

Whole cell lysates were charged with EBC cell lysates buffer (50 mM Tris pH8.0, 120 mM NaCl, 0.5% NP40, 0.1 mM EDTA and 10% Glycerol) containing protease inhibitor (B14001, Selleck) and phosphatase inhibitor (B15001, Selleck). Mitochondrial isolation kit from Beyotime (C3601). Protein concentration quantification using the BCA kit from Thermo Scientific (23227). Separation of proteins by SDS-PAGE. Rabbit antibody anti-HIF1A (36169S), anti-HA (3724S), anti-alpha Tubulin (3873S) were from Cell Signaling Technology. Rabbit antibody anti-Flag (20543-1-AP), anti-AIF (17984-1-AP), anti-Histone H3 (17168-1-AP), anti-PKM2 (25659-1-AP) were from Proteintech.

### 4.3 Plasmids

Full length FLAG or HA tagged MT2A or PKM2 were amplified by PCR with a FLAG tag with a 5′ primer that introduced a MluI site and a 3′ primer that introduced a BamHI site. The PCR products were digested with the corresponding enzymes and cloned into the pHAGE-puromycin vector. pHAGE-MT2A-C7A-HA, pHAGE-MT2A-C15A-HA, pHAGE-PKM2-Y105E-FLAG, pHAGE-PKM2-R399E-FLAG, pHAGE-PKM2-K422R-FLAG were constructed using Mut Express II Fast Mutagenesis Kit V2 (C214-01) from Vazyme. All plasmids have been verified by sequencing. Lentiviral shRNA target sequences are as follows:

MT2A shRNA-3: CGCCGGCTCCTGCAAATGCAA
MT2A shRNA-4: CCGGCTCCTGCAAATGCAAAG

### 4.4 Transfection and virus infection

Plasmids were transfected into cells using PEI (24765, PolyScience) at approximately 50% cell growth density and harvested 36 h after transfection. 293T cell line for lentiviral/retroviral packaging, Lentiviral/retroviral infection was performed as previously described (Zhang et al., 2018). Briefly, 293T cells were transfected with PEI, viruses were collected twice after 36 and 60 h. After passing through 0.45 μM filters, appropriate amount of viruses was used to infect target cells in the presence of 8 μg/mL polybrene (107689, SIGMA). Subsequently on the next day, the target cell lines underwent appropriate antibiotic selection.

### 4.5 Mitochondria isolation and proteinase K treatment

Mitochondria were extracted with Cell Mitochondria Isolation Kit (Beyotime Biotechnology, C3601) according to the manufacturers’ protocol. In brief, harvested cells were washed with cold PBS and disrupted mechanically with a Dounce homogenizer in cell mitochondria isolation reagent plus PMSF, and then incubated on ice for 15 min. The cell solutions were centrifuged at 1000*g* for 10 min at 4 °C, and the cell debris and nuclei were removed to obtain the supernatant. Mitochondrial fractions were pelleted by centrifugation at 12,000*g* for 20 min. The final supernatant was subjected to 16,000*g* centrifugation for 20 min at 4 °C to isolate cytosol. For Proteinase K assays, the freshly isolated mitochondria were incubated with the indicated concentrations of Proteinase K (Millipore, 3016611) for 30 min on ice, the reaction was terminated by PMSF (1 mM) and analyzed by immunoblot analysis (Yan et al., 2020).

### 4.6 Cell proliferation assays

Cells were seeded into 96-well (2500 cells/well) plates in the appropriate culture medium. At indicated time points, cells were replaced with 90 μL fresh growth medium supplemented with 10 μL 5 mg/mL 3-(4,5-dimethyl-2-thiazolyl)-2,5-diphenyl-2-H-tetrazolium bromide (MTT) (143315, BioFrox) per well, followed by incubation at 37 °C for 2 h. Then, this medium was removed completely and added 100 μL DMSO per well. Finally, the cell growth was measured by Microplate Reader at 490 nm wavelength.

### 4.7 Immunoprecipitation and mass spectrometry (IP-MS)

Cells were lysed in EBC lysis buffer supplemented with complete protease inhibitor and phosphatase inhibitor (Selleck). The cell lysate was ultrasonically broken and centrifuged at 16,000*g* for 20 min at 4 °C, and the supernatant was collected to remove cellular debris, and then mixed with primary antibodies or Flag-conjugated beads (Sigma, A2220) overnight at 4 °C. For primary antibody incubation overnight, cell lysates were further incubated with protein G sepharose beads (Thermo Fisher Scientific, 20399) for 4 h at 4 °C. Bound complexes were washed with NETN buffer six times and were eluted by boiling in SDS loading buffer. Bound proteins were resolved in SDS-PAGE followed by western blot analysis (Zhang et al., 2015).

To determine the MT2A-interacting proteins, MT2A-Flag and its interacting proteins were immunoprecipitated with Flag-beads from T47D cells (1×10^8^) stably expressing MT2A-Flag followed by LC-MS/MS, and the data were analyzed using Proteome Discoverer (v.2.2, Thermo Scientific).

### 4.8 Measurement of PKM2 activity

PKM2 activity was measured using the Human PKM2 ELISA Kit (H6063, Elabscience). Briefly, the adherent cells were washed once with PBS and then digested down with trypsin and centrifuged at 1000*g* for 5 min to obtain the cell pellets. The pellet was resuspended with PBS followed by sonication to break the cells, then centrifuged at 1500*g* for 10 min at 4 °C to obtain the supernatant which was assayed with the Kit.

### 4.9 Measurements of basal respiration and basal glycolysis

Basal respiration and basal glycolysis were measured by an XFe24 extracellular flux analyzer (Agilent Technologies), measurements according to the manufacturer's instructions. Briefly, 4×10^4^ T47D cells were seeded into XF24 cell culture microplate coated with CellTak (Corning) 12 h before the assay. Cells were washed twice with phenol red-free RPMI medium pH 7.4 (Agilent Technologies) supplemented with 10 mM glucose, 2 mM glutamine, 1 mM sodium pyruvate for basal respiration or 2 mM glutamine for basal glycolysis prior to experimentation. Cells were equilibrated within 1 h in 37 °C incubator without CO_2_. Then the basal respiration or basal glycolysis was recorded in response to sequential addition of indicated compounds from the Seahorse XF Cell Mito Stress Test Kit (Agilent Technologies, 103015-100) or Glycolysis Stress Test Kit (Agilent Technologies, 103020-100). The basal respiration and basal glycolysis values were normalized the amount of protein per well by BCA kit (Thermo Scientific) and analyzed using WAVE software (Agilent Technologies).

### 4.10 Real-time quantitative PCR

Total RNA was extracted from the cells using TRIzol reagent (15596018, Invitrogen) then reverse transcribed into cDNA using PrimeScript RT Master Mix (RR047A, TaKaRa). The cDNA was mixed with primers and qPCR SYBR Green Master Mix (Q111-2, Vazyme) and the reaction was detected using CFX Connect Real-Time PCR Detection System (Bio-Rad). Results were analyzed using method 2^-ΔΔCt^. The primers used were as follows:

MT2A-F: 5′- AAAGGGGCGTCGGACAAGTG-3′
MT2A-R: 5′-CAGCATCACAGTAAACCCGTGG-3′

### 4.11 Survival analysis

Survival analysis was performed using the R packages survival (v3.8-3) and survminer (v0.5.0) in R version 4.3.2. The analysis was based on 995 breast cancer patient cases from the METABRIC dataset. Samples were stratified into two groups according to the expression levels of the gene MT2A: the top 20% (high expression) and the bottom 20% (low expression). Kaplan-Meier survival curves were generated, and the log-rank test was applied to assess the statistical significance of survival differences between the two groups. The survival endpoints analyzed included overall survival (OS, defined as the time from diagnosis or treatment initiation to death from any cause), disease-free survival (DFS, defined as the time from the end of treatment to disease recurrence or death from any cause), and disease-specific survival (DSS, defined as the time from diagnosis or treatment initiation to death from the specific disease).

### 4.12 scRNA-seq analyses

Single-cell RNA sequencing (scRNA-seq) data from breast cancer patients were downloaded from the GEO database (GSE176078) and analyzed in this study. Data preprocessing, cell cluster annotation, and data integration methods were performed as described in previous literature. Specifically, cell filtering was conducted using the EmptyDrops method from the DropletUtils package (v1.2.2), with cells retained if they contained more than 200 genes, 250 unique molecular identifiers (UMIs), and less than 20% mitochondrial gene content. Malignant epithelial cells were identified using the inferCNV package (v0.99.7), with immune and endothelial cells serving as reference cells. Copy number variation (CNV) signals for individual cells were estimated, and epithelial cells were classified as normal, neoplastic, or unassigned based on genomic instability scores and correlation profiles. In this study, further analysis of malignant breast epithelial cells was conducted using the Seurat package (v4.4.0), focusing on expression differences across subtypes (ER positive, HER2 positive, TNBC). Hypoxia scores were calculated based on the Buffa gene set, and Response to Metal Ion pathway scores were derived from the corresponding GO pathway gene set, both using the AddModuleScore function for per-cell scoring. Correlation analysis was performed using the ggscatter function from the ggpubr package (v0.6.0) with the Pearson method.

### 4.13 Prediction of MT2A and PKM2 interaction by AlphaFold2

In this study, AlphaFold2 deployed on the computational cluster platform of the Medical Research Institute at Wuhan University was used to predict the structures of MT2A and PKM2 proteins, as well as their binding sites. AlphaFold2 generated five structural models for each protein, ranked according to pLDDT scores. The top-ranked model, with a structural confidence score of 0.908 (above the cutoff = 0.75), was selected for further analysis. The prediction of binding sites was performed by analyzing molecular interactions between MT2A and PKM2 proteins, with a focus on high-confidence regions (pLDDT >80). Specific analytical methods included selecting amino acids on MT2A and PKM2 with distances less than 5 Å and calculating the interaction forces present in the selected regions. The predicted protein structures and binding sites were visualized using PyMOL 2.6 to further validate and analyze their interaction patterns.

### 4.14 In vitro protein cross-linking experiment

293T cells transiently transfected with MT2A-HA were cultured under hypoxic conditions for 24 h. Then extract mitochondria using Cell Mitochondria Isolation Kit (Beyotime Biotechnology, C3601). Subsequently, 2 μg His-PKM2 protein be added to the mitochondrial solution, after which the mixture is to be incubated under hypoxic conditions for 1 h. After incubation, cross-link with 0.001 (*V*/*V*) glutaraldehyde at 37 °C for 9 min under hypoxic conditions. The reactions were terminated by adding 1M Tris-HCl buffer to a final concentration of 100 mM Tris-HCl (PH 8.0). The samples were separated on SDS-PAGE and analyzed by western blotting.

### 4.15 Mouse orthotopic tumor growth

Four-week-old female nude mice were purchased from GemPharmatech and used for our xenograft studies (*n* = 6/group). Each mouse was injected in situ into the mammary gland with 1×10^6^ MDA-MB-231 cells mixed with Matrigel Matrix (354234, CORNING) as described previously (Zhang et al., 2009). Measured the size of the tumors every three days with a vernier caliper. After 27 days, all mice were euthanized, and the tumors were excised and weighed. Mice were bred and maintained in Animal Center of Medical Research Institute at Wuhan University. All animal experiments were performed according to protocols approved by the Animal Care and Use Committee of Medical Research Institute, Wuhan University.

### 4.16 Statistical analysis

All error bars represent SD, two-tailed Student *t*-test (∗, *P* < 0.05; ∗∗, *P* < 0.01; ∗∗∗, *P* < 0.001; ns, denotes no significance). Data statistics using GraphPad Prism software version 8.0.2.

## CRediT authorship contribution statement

**Ronghui Gao:** Writing – original draft, Methodology, Investigation, Formal analysis, Data curation. **Qifang Li:** Validation, Methodology, Investigation, Formal analysis, Data curation. **Jiahao Guo:** Methodology, Formal analysis, Data curation. **Zirou Peng:** Investigation, Formal analysis, Data curation. **Chuan Gao:** Formal analysis, Data curation. **Zirui Zhou:** Investigation. **Hankun Hu:** Writing – review & editing, Funding acquisition. **Jing Zhang:** Writing – review & editing, Supervision, Resources, Project administration, Funding acquisition, Conceptualization.

## Declaration of competing interest

The authors declare that they have no known competing financial interests or personal relationships that could have appeared to influence the work reported in this paper.
